# Checking the STEP-Associated Trafficking and Internalization of Glutamate Receptors for Reduced Cognitive Deficits: A Machine Learning Approach-Based Cheminformatics Study and Its Application for Drug Repurposing

**DOI:** 10.1371/journal.pone.0129370

**Published:** 2015-06-12

**Authors:** Salma Jamal, Sukriti Goyal, Asheesh Shanker, Abhinav Grover

**Affiliations:** 1 Department of Bioscience and Biotechnology, Banasthali University, Tonk, Rajasthan, India; 2 School of Biotechnology, Jawaharlal Nehru University, New Delhi, India; Aligarh Muslim University, INDIA

## Abstract

**Background:**

Alzheimer’s disease, a lethal neurodegenerative disorder that leads to progressive memory loss, is the most common form of dementia. Owing to the complexity of the disease, its root cause still remains unclear. The existing anti-Alzheimer’s drugs are unable to cure the disease while the current therapeutic options have provided only limited help in restoring moderate memory and remain ineffective at restricting the disease’s progression. The striatal-enriched protein tyrosine phosphatase (STEP) has been shown to be involved in the internalization of the receptor, N-methyl D-aspartate (NMDR) and thus is associated with the disease. The present study was performed using machine learning algorithms, docking protocol and molecular dynamics (MD) simulations to develop STEP inhibitors, which could be novel anti-Alzheimer’s molecules.

**Methods:**

The present study deals with the generation of computational predictive models based on chemical descriptors of compounds using machine learning approaches followed by substructure fragment analysis. To perform this analysis, the 2D molecular descriptors were generated and machine learning algorithms (Naïve Bayes, Random Forest and Sequential Minimization Optimization) were utilized. The binding mechanisms and the molecular interactions between the predicted active compounds and the target protein were modelled using docking methods. Further, the stability of the protein-ligand complex was evaluated using MD simulation studies. The substructure fragment analysis was performed using Substructure fingerprint (SubFp), which was further explored using a predefined dictionary.

**Results:**

The present study demonstrates that the computational methodology used can be employed to examine the biological activities of small molecules and prioritize them for experimental screening. Large unscreened chemical libraries can be screened to identify potential novel hits and accelerate the drug discovery process. Additionally, the chemical libraries can be searched for significant substructure patterns as reported in the present study, thus possibly contributing to the activity of these molecules.

## Background

Alzheimer’s disease is a major public health problem globally and contributes to 60–80% of all dementia cases. Alzheimer’s disease (AD) is a progressive neurodegenerative disorder that predominantly affects the elderly population [1]. According to the World Alzheimer Report 2013, AD is the sixth leading cause of mortality in USA and currently affects some 35 million people worldwide. The report states that the annual number of new cases of AD will double by 2030 and triple by 2050 [2]. The disease is characterized by progressive memory loss, impairment of cognitive domains such as language, and mood disturbances. The exponential rise in the number of patients with AD along with the socioeconomic burden associated with it has made it necessary to discover novel drugs for the treatment of the disease [3].

The US Food and Drug Administration has approved five drugs, which are currently used for the treatment of AD, although none of them have been able to curtail or hamper the disease’s progression. The currently available therapeutic options for AD include: three cholinesterase inhibitors, namely Donepezil, Rivastigmine and Galantamine, and one N-methyl D-aspartate antagonist (i.e. Memantine). The drugs presently available are not effective to any significant degree and their effectiveness varies according to the population. The major difficulty in the treatment of AD is the narrowing therapeutic options. This has made it necessary to develop effective and secure drugs, which can reduce the overall burden of the disease [4, 5].

Various hypotheses have been proposed regarding the cause of AD. However, the characteristic abnormalities include the aggregation of amyloid-β (Aβ) plaques and tau protein tangles in the brain. Aβ is a short peptide resulting from the amyloid precursor protein (APP), which undergoes various conformational changes and aggregates to form plaques outside the brain [6]. The soluble Aβ forms result in the loss of synaptic functions as well as synapse and cognitive impairment [7, 8]. Striatal-enriched protein tyrosine phosphatase (STEP), a brain-specific protein tyrosine phosphatase, preferably expressed in the cortex, hippocampus and related brain structures, regulates the trafficking of NMDRs (N-methyl D-aspartate receptors) [9]. The STEP associates with the NMDRs, a class of glutamate receptors, and lessens their activity by dephosphorylating the tyrosine (Tyr), which leads to the internalization of NMDRs in the brain. The over-expression of STEP leads to excessive trafficking of glutamate receptors, NMDRs, which has been related to the synaptic changes in the brain that ultimately lead to neurodegenerative conditions such as AD [10].

In the present study, we have generated machine learning based models using the high-throughput bioassay available on PubChem [11] that was developed to identify inhibitors of STEP. The assay conducted is based on the hypothesis that STEP inhibitors may reduce the cognitive deficits in the brain and thus may prove therapeutic in terms of slowing down disease progression. The work carried out in this study comprises preprocessing with molecular descriptors followed by machine learning based classification, subsequent structure based molecular docking and processing with molecular dynamics simulation.

## Computational Methodology

### Data Source of the Inhibitors

A total of 359,231 striatal-enriched protein tyrosine phosphatase (STEP) inhibitors and non-inhibitors were downloaded from PubChem, which is a large repository of chemical structures along with their biological assay activities. The fluorescence-based bioassay provided at PubChem with assay id: 588621 to identify the small molecule inhibitors of STEP was used in the study. According to the assay, the activity score of the compounds was reported at a 20 microMolar concentration. The compounds showing > = 40% inhibition activity at a 20 microMolar concentration were considered as active compounds, while the rest were categorized as inactive. The actives were assigned a bioassay activity score of 20 while the inactives were assigned a score of 0. The screening classified 887 compounds as active and 358,344 as inactive.

### Molecular Descriptors Calculation

The molecular structures, downloaded in Structural Data Format (SDF), were converted into a vector of features known as descriptors using the freely available PowerMV [12] software. PowerMV calculated a total of 179 2D molecular descriptors, which comprise 147 Pharmacophore fingerprints, 24 weighted burden number descriptors and 8 property descriptors. The descriptors found to be redundant throughout the data were removed using the RemoveUseless filter implemented in Weka (version 3.6.11) [13], a machine learning suite of programs.

### Descriptor Selection Method

It has been reported that excessive descriptors may lead to over fitting of the model and so increase the dimensionality of the dataset in addition to the computational time involved. Not all descriptors are relevant to the data classification and thus irrelevant descriptors were removed using various feature selection methods in order to generate robust classification models [14, 15]. Feature selection techniques reduced the noise from the data by decreasing the number of features, concentrating on the significantly contributing subset and speeding up the data mining process along with improving the resulting accuracy of the classifiers.

In the present study, we have used the CfsSubsetEval module in combination with the BestFirst attribute selection algorithm integrated in Weka to select relevant descriptors. CfsSubsetEval considers the predictive ability of each descriptor in addition to the redundancy among the features and puts forward a subset of descriptors that are highly correlated with the discriminating class but have low inter-correlation. The BestFirst search algorithm searches the space of attributes by greedy hill-climbing along with a backtracking facility by searching in both forward and backward directions. Initially, a subset of the best features is chosen randomly and a new feature is obtained based on information gained from the features of the chosen set. Subsequently, the initially chosen descriptors are removed and the process is repeated until all the features have been considered at least once.

### Machine Learning Based Classification Models

We have used a set of machine learning algorithms, namely the Bayes theorem-based Naive Bayes (NB), the decision tree-based Random Forest (RF) and the support vector machine-based Sequential Minimization Optimization (SMO), for the prediction of STEP inhibitors and non-inhibitors.

The Naive Bayes classifier performs by computing the probability of a compound being active or inactive and then assigning that compound to a class with maximum probability. While calculating the probability, the classifier assumes all the attributes to be independent of each other [16]. NB classifier has the advantage of being the simplest and the fastest classifier and thus has already been used quite frequently in a number of studies [17, 18]. The model was generated keeping all the parameters as default in Weka (S1 table).

The Random Forest classification algorithm is based on a group of random decision trees constructed using multiple features for each molecule from the training set. The final class that a molecule is assigned to is determined by the mode of the decision of the individual trees (i.e. the mode of the output class of the individual trees) [19]. RF uses an ensemble of decision trees to assign a specific class to a particular molecule and previous studies have shown that the multiple decision trees give much accurate prediction than an individual tree [20]. Default parameter settings were maintained in Weka while running the Random Forest classifier (S1 table).

Sequential Minimization Optimization is a support vector machine-based classification algorithm, which uses a kernel function, constructs a hyperplane and then attempts to maximize it to find best possible separation between the two classes [21]. SMO has the ability to handle very large datasets by breaking the large problem into many small problems and then solving each small problem which makes it the most preferred machine learning classifier [22, 23, 24, 25]. PolyKernel was used and the ‘Build logistic models’ was set true, while all the other parameter settings were kept as default in Weka (S1 table).

These classifiers have already been explained in detail elsewhere [16, 19, 21]. In the present study, the classification was conducted using a machine learning tool, Weka (version 3.6.11) [13], which provides a set of machine learning algorithms for data mining experiments. The results obtained using the various classification algorithms were then compared. The models were generated using descriptors at two different levels: one using the descriptors obtained by using the RemoveUseless filter of Weka and the other with the descriptors obtained using the CfsSubsetEval module.

### Handling Imbalanced Data

One of the principle problems with high-throughput screen datasets is the imbalance in data (i.e. the presence of one class as a majority compared to another class) [26]. In the bioassay dataset used in the present study, the number of active compounds was 887, making it the minority class, while the number of inactive compounds was 358,344, making it the majority class. This could result in a biased classification by the classifier, which may predict every compound as belonging to the major class and therefore overlook the minority class.

Weka handles misclassification problems due to a class imbalance by using cost sensitive classification and implementing it using a 2*2 confusion matrix. The 2*2 cost matrix consists of four segments: true positives (TP), true negatives (TN), false positives (FP) and false negatives (FN). In cost sensitive classification, the misclassification costs are applied, although the algorithm then tries to minimize them. Misclassifying an active molecule cannot be allowed and thus the costs are applied to false negatives to reduce their number and retain active compounds. However, reducing the number of false negatives simultaneously increases the false positives. Therefore, a threshold of 20% was established and the cost of false negatives was increased until the false positives rate reached 20%. The threshold value used in this study has been determined empirically as satisfactory results have been reported in a plethora of previous studies [26, 27, 28] where this 20% false positive threshold has been used.

### Evaluation of Performance

The predictive performance of the classifiers was evaluated using a cross validation approach where an internal cross validation was conducted on the training set. During cross validation, the training data was divided into n subsets, with the n-1 subsets being used as the train set and the left out single subset being used as the test set. The process was repeated n times and each left out single subset was used once as the validation set on the trained model. The performance of the machine learning methods can be assessed in terms of the four elements of the confusion matrix, which include true positives (TP), false positives (FP), true negatives (TN) and false negatives (FN). Various statistical measures for assessing sensitivity, specificity the overall accuracy and G-mean, were used to measure the performance of the classifiers in determining inhibitors from non-inhibitors. A receiver operating characteristic (ROC) plot was also used to evaluate the performance of the generated models by computing the Area under Curve (AUC).

Sensitivity=TP/TP+FN

Specificity=TN/TN+FP

Accuracy=TP+TN/TP+TN+FP+FN

$$\text{G-mean}=\sqrt{Sensitivity*Specificity}$$

### SMART Filtering of the Data

The SMART pattern represents the unwanted fragments present in the compounds that have been found to be toxic or to have undesirable effects. The compounds predicted to be active by the machine learning model were passed through the SMARTsfilter (http://pasilla.health.unm.edu/tomcat/biocomp/smartsfilter) web application to filter out the compounds containing any of the SMART patterns. The SMARTs set includes five filters, namely PAINS [29], Glaxo [30], ALARM NMR [31], Pfizer LINT and Oprea. The compounds were passed through each of the five filters and any compound that matched any of the SMART set was considered as having failed, while those compounds that did not match to any of the five SMARTs were categorized as having passed.

### Molecular Docking

The X-ray crystal structure of the human protein tyrosine phosphatase, PTPN5 (STEP, striatum enriched phosphatase) (PDB ID: 2BV5), was downloaded from the protein data bank (PDB) [32] at a resolution of 1.8 Å. The protein was further pre-processed and optimized using the Protein Preparation Wizard implemented in Schrodinger Suite [33, 34]. Using the Receptor Grid Generation panel of Schrodinger, the grid was generated using the centroid of selected residues, Ser473, Ala474, Gly477 and Arg478, taking the radius as 20 Å. The scaling factor and partial charge cut off were kept as default at 1.0 and 0.25, respectively, and no constraints were defined. The compounds that passed the SMARTsfilter were further screened using the docking approach and were prepared using Schrodinger’s LigPrep [35] software.

The prepared ligands were subjected to docking within the active site of STEP using Schrodinger’s Glide (Grid-based Ligand Docking with Energetics) [36, 37] module. Glide performs an extensive conformational, locational and orientational search over the active site of the protein for each ligand and generates an output of Glide GScore and Glide energy. The Glide GScore includes Van der Waals energy, Coulomb energy, hydrophobic interactions, hydrogen bonds, and polar interactions in the active site and solvation terms. The ligands were first docked using the High-Throughput Virtual Screening (HTVS) docking protocol of Glide, following which the top scoring resulting ligands were further subjected to Glide’s Extra Precision (XP) docking strategy. The final top scoring compounds were selected based on the Glide GScore and Glide energy.

### Molecular Dynamics Simulation Studies of the Docked Complex

The molecular dynamics (MD) simulation studies were performed using the Desmond Molecular Dynamics system implemented in Schrodinger [38]. The MD studies were performed to analyze the stability of the docked ligand-protein complex. Prior to the MD simulation, the docked complex was prepared using Schrodinger’s Protein Preparation wizard. The prepared protein was then solvated using a simple point charge (SPC) solvated model in an orthorhombic boundary box with a 10 Å distance between the atoms of the protein and the sides of the box. The force field used was the Optimized Potentials for Liquid Simulations (OPLS) all-atom force field 2005 [39, 40]. Subsequent to the system builder step, the solvated protein was minimized using the steepest descent (SD) minimization algorithm, which carried out a maximum of 5000 steps until a gradient of 25 kcal/mol/Å was reached. This was followed by another minimization using the Limited-memory Broyden Fletcher Goldfarb Shanno (LBFGS) algorithm and a convergence criteria of 1 kcal/mol/Å. The minimized system was then used to perform the dynamics study for which simulations were carried out in the Isothermal-Isobaric ensemble (NPT) at 300 K temperature and 1 atm pressure. During the minimization stages in MD simulation, the initial minimization was performed with restraints on solute, followed by another minimization without any restraints. Restraints applied only on the heavy atoms of the solute were used throughout the simulation stages, however no restraints were applied in the final stage of the MD simulations study. Constraints are implemented using M-SHAKE algorithm in Desmond. The two parameters, maximum iteration count, m, which is the number of iterations to be used in the constraint involved was set to 8 and relative tolerance, δ, for the constraint algorithm was set to was set to 10–8. The simulation for the docked complex was carried out for 15 ns and the Smooth Particle Mesh Ewald Method was used to handle long range electrostatic interactions. A cut off method with a default value of 9 Å as the cut off was used for handling the short range interactions. The reference system propagator algorithms (RESPA) were used with a 2 fs time step for bonded interactions, 2 fs time step for near-bonded and 6 fs time step for far-bonded interactions. Further, the root mean square deviation (RMSD) was computed and analyses were performed for all the trajectories in the 15 ns MD simulation run.

### Substructure Fragments

The inhibitors and non-inhibitors were searched for the presence of structural fragments using the same substructure pattern recognition method as that used by Shen et al. [41] and Raghava et al. [15]. The freely available PaDEL [42] software was used to generate substructure fingerprints (SubFP), which generated a total of 307 substructure patterns. These substructures were further searched in both the actives and inactives and were analyzed using the predefined substructure dictionary available in PaDEL. The frequency of the substructure fragments in the inhibitors and non-inhibitors was calculated using the following formula:

$$\mathrm{Frequency of a fragment}=\left(N_{fragment\_class}*N_{total}\right)/\left(N_{fragment\_total}*N_{class}\right)$$

where N_fragment_class_ is the number of compounds containing the substructure fragment in a STEP inhibition class, N_total_ is the total number of compounds, N_fragment_total_ is the total number of compounds containing the fragment, and N_class_ is the number of compounds in the inhibition class.

## Results and Discussion

The present study was carried out to identify inhibitors of striatal-enriched phosphatase (STEP) enzyme, which may prove therapeutic for Alzheimer's disease (AD). The high-throughput assay used in the study was conducted on a total of 359,231 compounds using fluorogenic phosphatase substrate and resulted in a total of 887 hit compounds that were categorized as actives while the remaining 358,344 were considered as inactive compounds.

### Molecular Descriptors Analysis

The 179 2D molecular descriptors calculated using PowerMV were processed using the RemoveUseless filter in Weka. The resultant 154 attributes included 122 Pharmacophore fingerprints, 24 weighted burden number descriptors and 8 property descriptors. Further, the BestFirst search algorithm of Weka was applied on the non-redundant set of 154 descriptors. The BestFirst search selected a set of 10 descriptors, which included 6 Pharmacophore fingerprints and 4 weighted burden number descriptors. The list of the 154 non-redundant attributes and 10 BestFirst descriptors is given in S2 table.

### Performance of the Machine Learning Models

The machine learning models were generated using both sets of descriptors: all 154 descriptors and 10 BestFirst descriptors using the classification algorithms NB, RF and SMO. The analysis was performed using a 5-fold cross validation. S3 table lists the performance of the classifiers using 5-fold cross validation.

**Fig 1:**
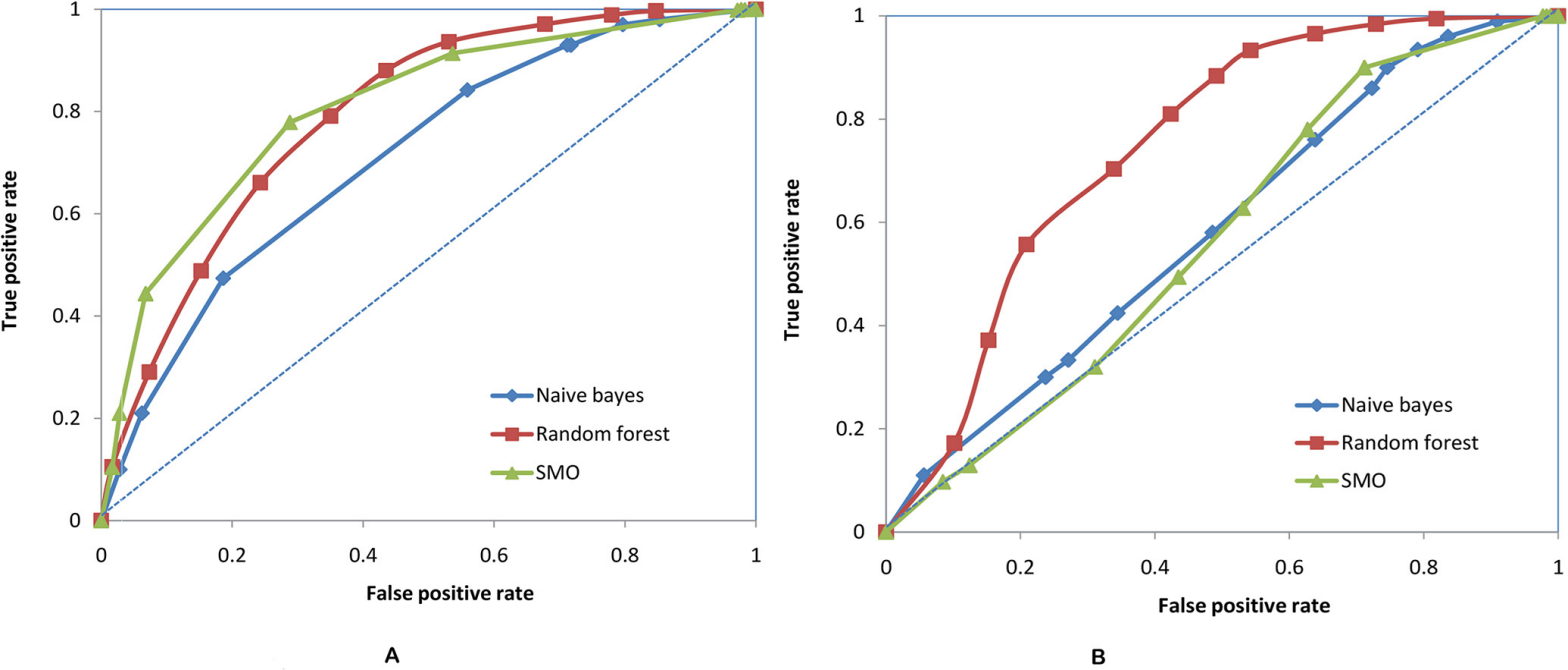
ROC plot for the three machine learning models generated using (A) 154 descriptors. (B) 10 BestFirst descriptors.

All the models using 154 descriptors correctly predicted >75% of the test set compounds. NB was found to be the least sensitive, with a sensitivity of 52%, while RF and SMO showed a sensitivity of 64.9% and 69.4% respectively. All the models showed around 79% specificity. However, since sensitivity and specificity do not provide a balanced classification, the G-mean was calculated. SMO performed the best and had a G-mean value of 0.74 (Table 1). NB had an AUC value of 0.72, RF had an AUC value of 0.79 and SMO had a value 0.81 (Fig 1(A)).

**Table 1:** Evaluation of generated models based on 154 attributes obtained using RemoveUseless filter.

Models	True positives	True negatives	False positives	False negatives	Sensitivity (%)	Specificity (%)	Accuracy (%)	G-mean
*NB*	92	57215	14454	85	52	79.8	79.7	0.64
*RF*	115	56697	14972	62	64.9	79.1	79	0.71
*SMO*	123	56903	14766	54	69.4	79.3	79.3	0.74

The models based on the BestFirst descriptors showed better accuracy and a specificity of around 80%. However, they also showed a high rate of false positives. NB was again found to be the least sensitive (Sn = 32.8%), followed by SMO (Sn = 36.7%), with RF being 57.6% sensitive. RF performed best in terms of G-mean value at 0.68 (Table 2). The AUC values from the ROC plot are 0.58, 0.74 and 0.57 for NB, RF and SMO respectively (Fig 1(B)).

**Table 2:** Evaluation of generated models based on 10 BestFirst attributes obtained using CfsSubsetEval module.

Models	True positives	True negatives	False positives	False negatives	Sensitivity (%)	Specificity (%)	Accuracy (%)	G-mean
*NB*	58	57588	14081	119	32.8	80.3	80.2	0.51
*RF*	102	58049	13620	75	57.6	80.9	80.9	0.68
*SMO*	65	57796	13873	112	36.7	80.6	80.5	0.54

Since the models based on both levels of descriptors showed similar accuracy, the RF model based on the BestFirst descriptors was used for further analyses owing to its higher accuracy.

### SMARTs Analysis

**Fig 2:**
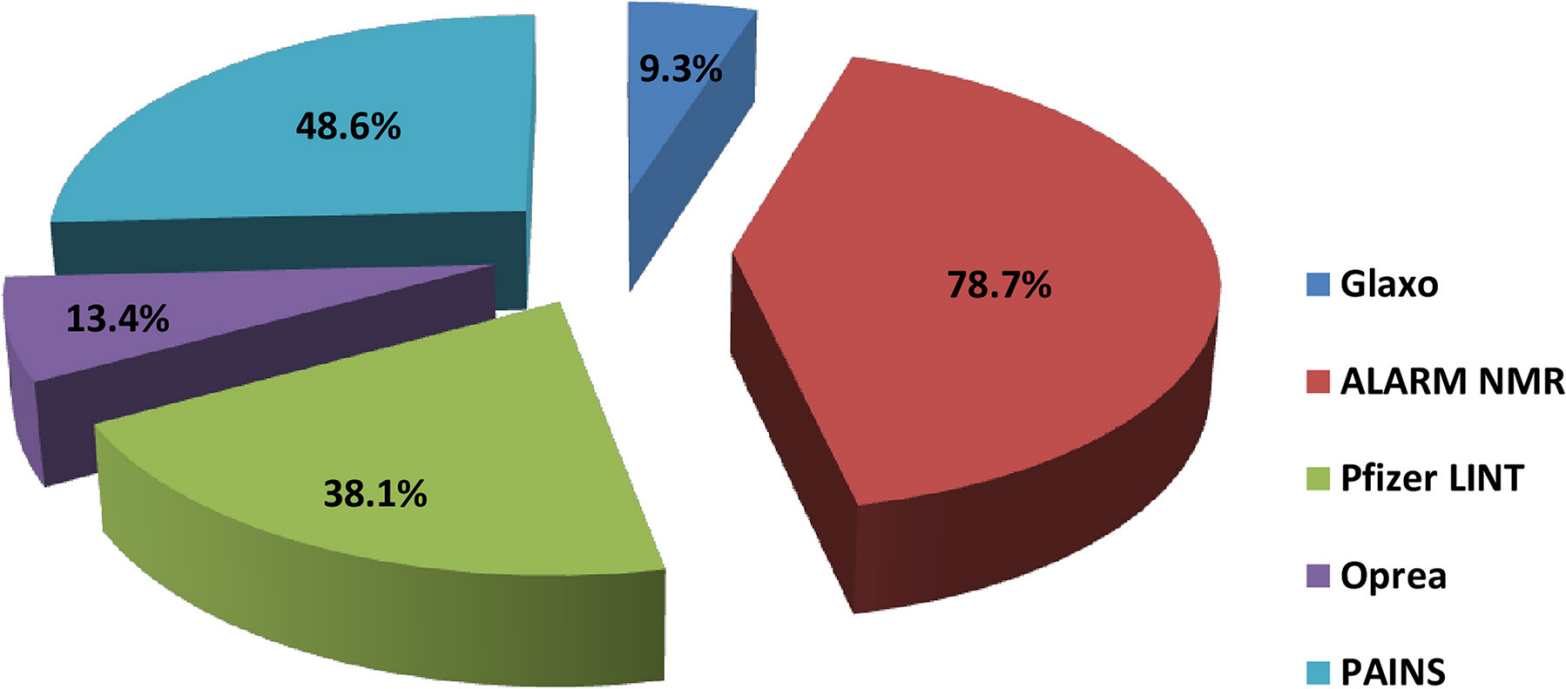
shows the %age of molecules filtered using SMARTs filter.

We analyzed the test instances predicted to be active by our best model for the presence of SMART patterns to filter out unwanted molecules. A total of 13,722 predicted positives were processed through the SMARTsfilter web application and 1,299 molecules passed through all the filters, confirming the absence of any SMART pattern (Fig 2). The results indicated that the majority of compounds (i.e. 78.7%) could not pass the ALARM NMR filter followed by the PAINS (48.6%) and Pfizer LINT (38.1%). Some 13.4% and 9.3% of compounds could not pass Oprea and Glaxo filters respectively. We also examined the 887 active compounds of the bioassay, of which only 30 compounds (3.4%) passed through all the five sets of SMARTs.

### Molecular Docking Analyses

**Fig 3:**
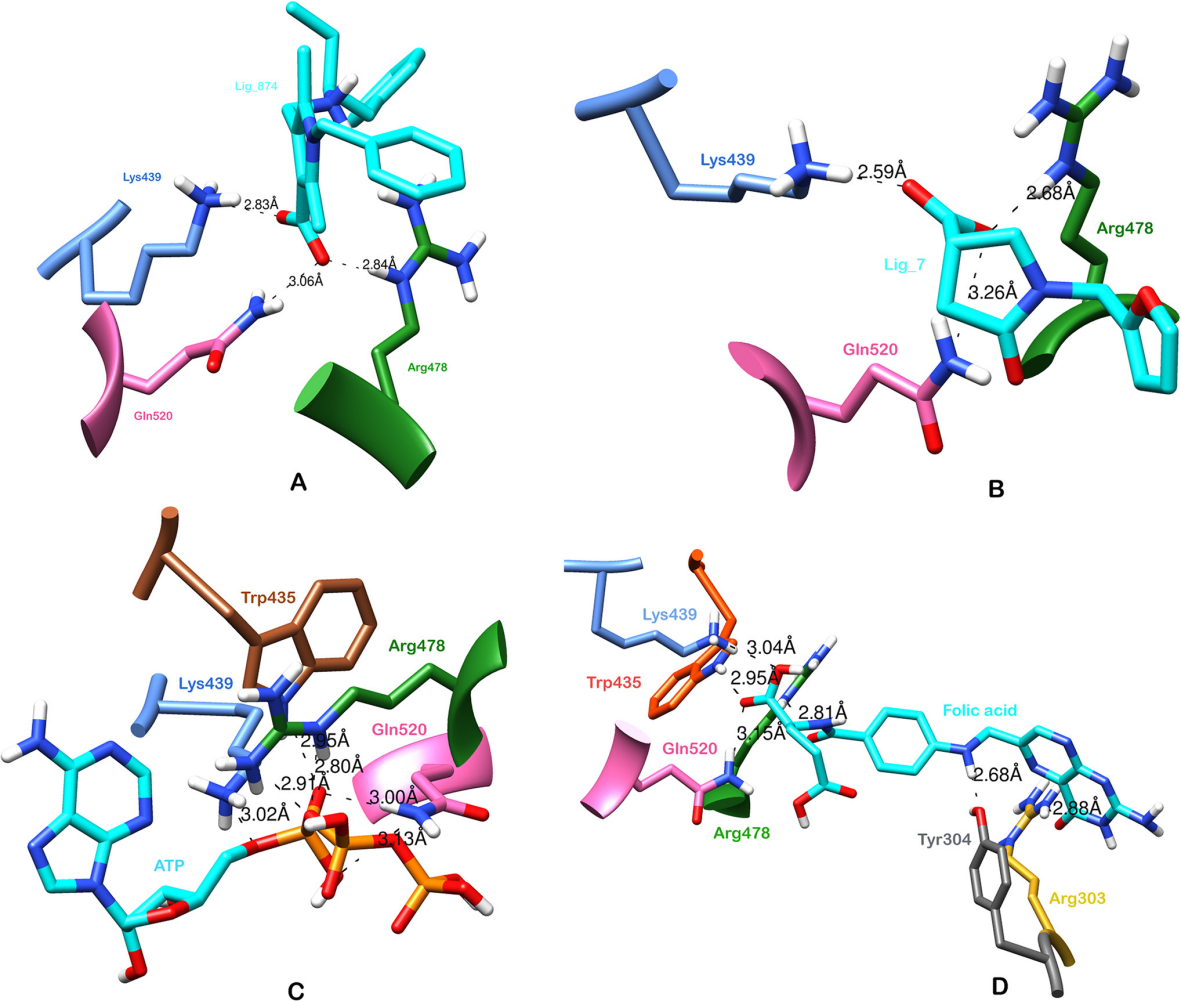
Docked conformations of the lead compounds with STEP protein (A) H-bond interactions of Ligand_7 (B) H-bond interactions of Ligand_5 (C) H-bond interactions of ATP (D) H-bond interactions of Folic acid.

**Fig 4:**
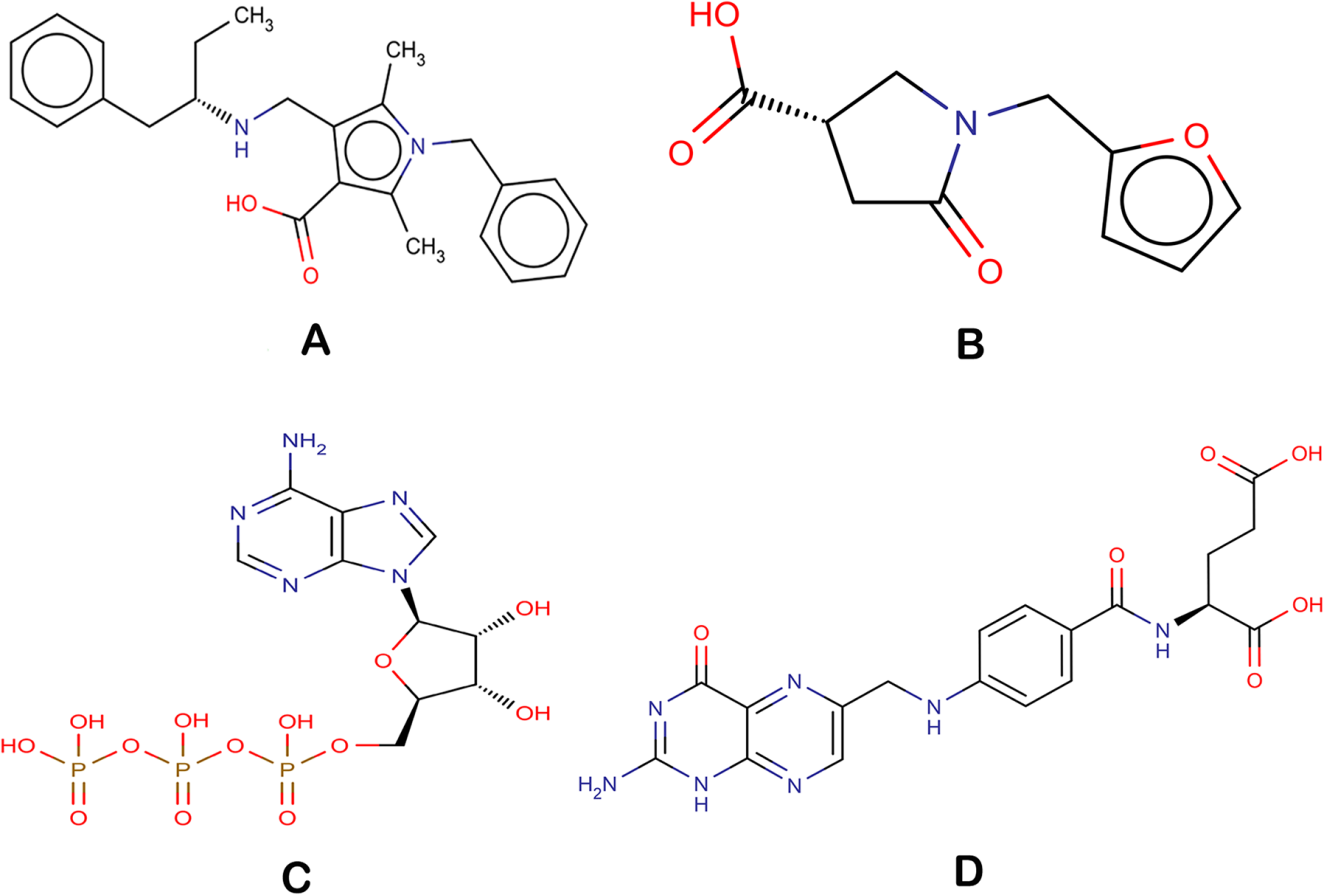
Chemical structures of the docked compounds (A) Ligand_7 (B) Ligand_5 (C) ATP (D) Folic acid.

The 1,299 molecules that passed the SMARTsfilter were also virtually screened against human protein tyrosine phosphatase, PTPN5 (STEP, striatum enriched phosphatase) (PDB ID: 2BV5), to further identify and rank the potential active molecules. Initially, the compounds were docked using the HTVS approach, which led to 49 compounds with a Glide score of less than -6.0 kcal/mol. The 49 compounds were then subjected to Glide XP docking. The top scoring compound (1-benzyl-2, 5-dimethyl-4-({[(2S)-1-phenylbutan-2-yl] azaniumyl} methyl)-1H-pyrrole-3-carboxylate, Ligand_874) among the resultant compounds had a Glide score of -8.11 kcal/mol and a Glide energy of -42.44 kcal/mol (Table 3, Fig 3(A)). The ligand_874 that occupied the active site defined and formed three hydrogen bonds with residues, Lys 439, Arg 478 and Gln 520. One hydrogen bond of 2.84 Å was formed between the NE atoms of Arg 478 amino acid, the residue involved in the active site, and the O2 atom of ligand_874. Two other hydrogen bonds were formed between the NZ of Lys 439 and the O1 of ligand_874 (2.83 Å) and the NE2 of Gln 520 and the O2 of ligand_874 (3.06 Å). The ligand was also observed to have many hydrophobic contacts involving the residues Tyr 304, Asn376, Glu378, Glu379, Trp435, Pro436, Asp437 and Gln516. The compound, ligand_874, also passed the Lipinski filter with a molecular weight of 390.52 g/mol, two donor hydrogen bonds, 3.5 acceptor hydrogen bonds and a partition coefficient (log P) value of 3.93. The interactions as well as the predicted physical properties suggest the compound (1-benzyl-2, 5-dimethyl-4-({[(2S)-1-phenylbutan-2-yl] azaniumyl} methyl)-1H-pyrrole-3-carboxylate, Ligand_874) to be a very promising candidate to fight Alzheimer’s disease. The chemical structure of the ligand is shown in Fig 4(A).

**Table 3:** Binding affinity scores, energies, molecular interactions as well as H-bond distances of the docked compounds.

Compound	Glide score (XP) (kcal/mol)	Interacting residues	Distance (H-bond, Å)	Glide energy (kcal/mol)
*Ligand_874* (1-benzyl-2, 5-dimethyl-4-({[(2S)-1-phenylbutan-2-yl] azaniumyl} methyl)-1H-pyrrole-3-carboxylate, Ligand_874) (AID 588621 dataset)	-8.11	Lys439, Arg478, Gln520, Tyr 304, Asn376, Glu378, Glu379, Trp435, Pro436, Asp437, Gln516	2.82, 2.84, 3.06	-42.44
*Ligand_7* (Sigma-Aldrich compounds)	-5.95	Lys439, Arg478, Gln520, Tyr 304, Trp435, Scy472, Gln516	2.59, 2.68, 3.26	-32.80
*Adenosine triphosphate (ATP)*	-9.34	Lys439, Arg478, Gln520, Trp435, Asn376, Pro436, Asp437, Scy472, Ser473, Gly477, Gln516	3.01, 2.80, 2.90; 2.99, 3.13; 2.94	-53.39
*Folic Acid*	-6.03	Lys439, Arg478, Gln520, Trp435, Arg303, Tyr304, Glu382, Scy472, Ser473, Gln516, Thr517	3.03, 2.81, 3.15, 2.95, 2.87, 2.68	-50.97

### Molecular Dynamics Simulation Studies of the Docked Complex

**Fig 5:**
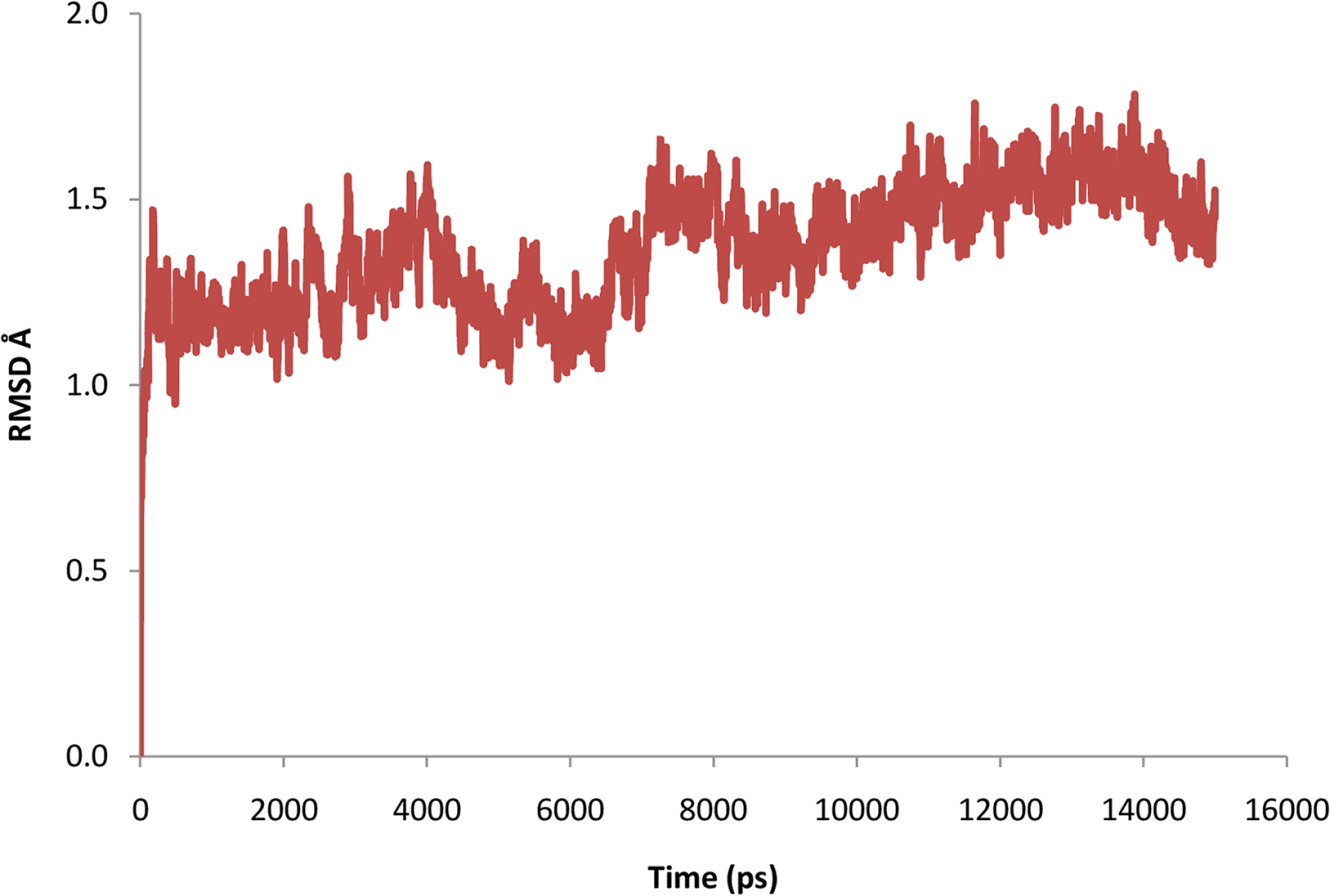
RMSD trajectory of the backbone obtained after MD simulation study.

**Fig 6:**
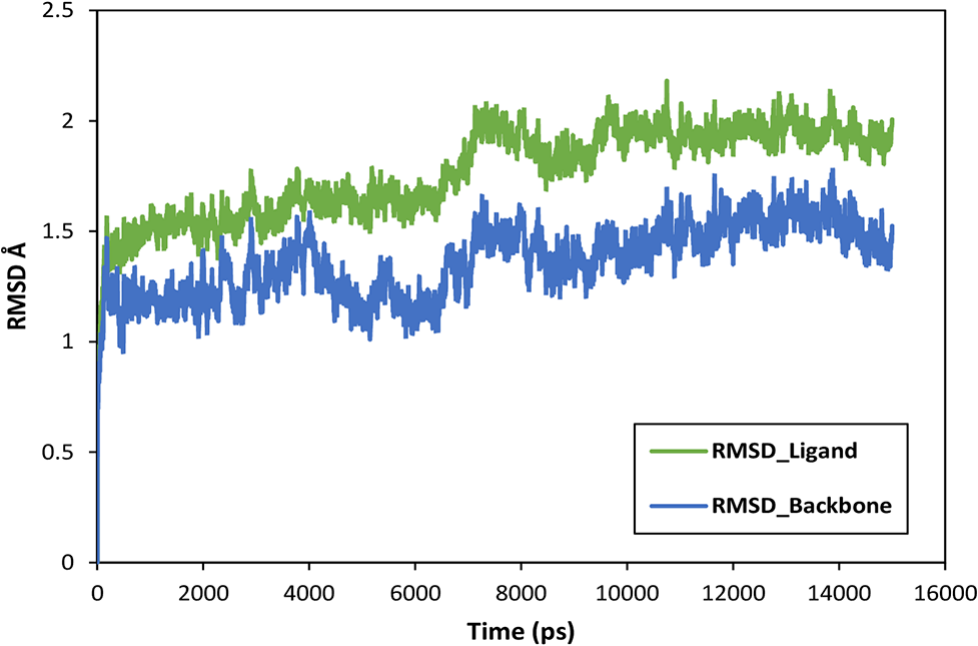
RMSD trajectory of the protein-ligand complex obtained after MD simulation study.

**Fig 7:**
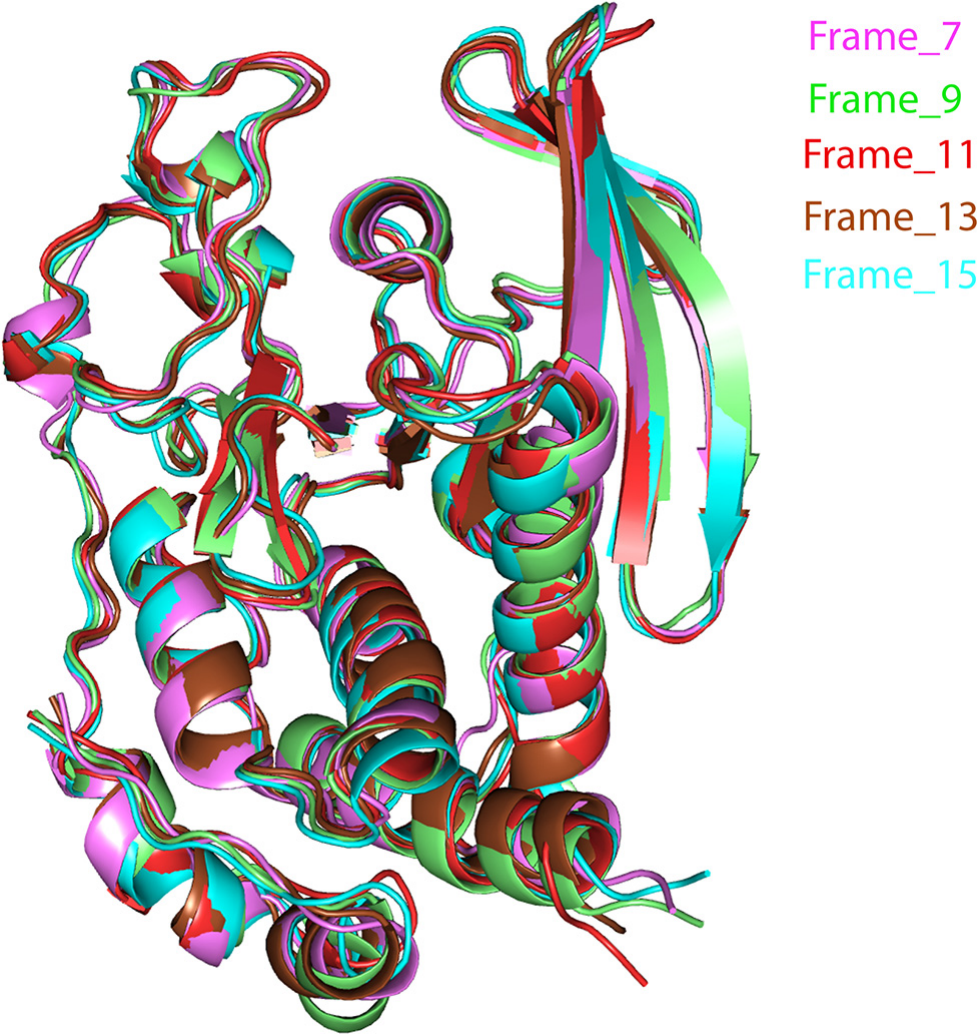
Superimposition of the structures of the complexes corresponding to various frames, 7, 9, 11, 13 and 15, during the trajectory analysis.

**Fig 8:**
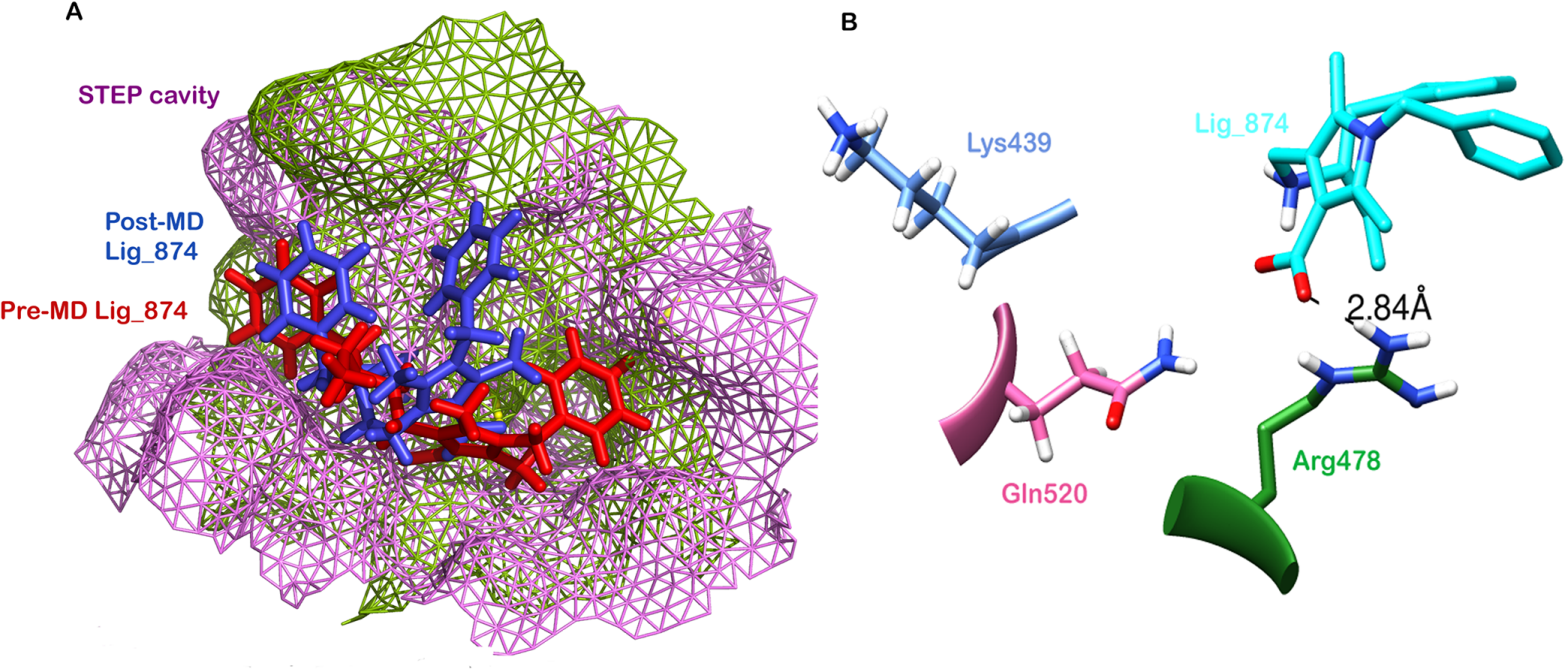
MD simulations (A) Superimposition of pre-MD (purple) and post-MD (green) complex of Ligand_7 with STEP, (B) H-bond interactions present in Ligand_7 with STEP complex obtained after MD.

**Fig 9:**
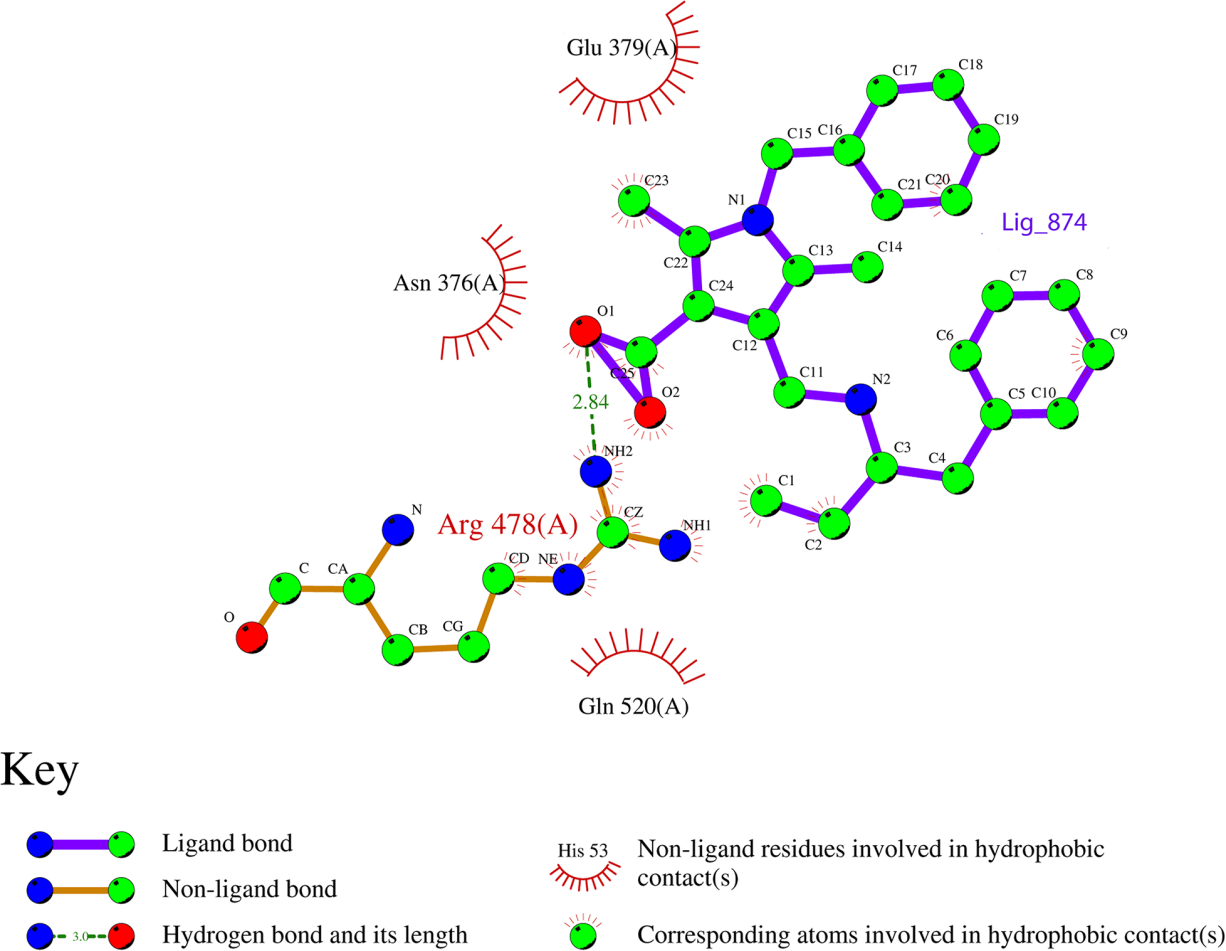
shows the hydrogen and hydrophobic interactions between the ligand and the protein.

To further evaluate the stability of the docked complex, the MD simulation of the Ligand_874 complex docked into the active site of STEP was carried out using the Desmond Molecular Dynamics system implemented in Schrodinger. As evident from Fig 5, the complex (backbone) acquired stability after 7 ns with a root mean square deviation (RMSD) of about 1.5 Å. The authors have computed the root mean square deviation (RMSD) of the ligand and the protein-ligand complex in addition to the RMSD of the backbone. The ligand alone was stable after 7 ns with a RMSD value of around 2 Å, and the backbone acquired stable trajectory beyond 7 ns with a RMSD value of ~1.5 Å. During the RMSD analysis of the protein-ligand complex, both the protein and the ligand acquired stability after 7ns having RMSD of about 1.5 Å and 2 Å respectively. As evident from Fig 6, RMSD plots of the protein and ligand alone and the RMSD plot of the full protein-ligand complex were clearly overlapping indicating that the complex was stable and no large scale conformational changes were found to be associated with the binding of the ligand. Additionally, we also extracted the structures of the simulated protein-ligand complexes corresponding to various frames over the entire trajectory and performed superimposition analysis of these structures in order to gain insights into the dynamic stability of binding (Fig 7). Fig 8(A) shows the superimposition of the ligand in the pre- and post-MD simulated structures of the docked complex. The complete MD simulation study was carried out for 15 ns, during which the Ligand_874 lost all the interactions formed in the pre-MD docked pose, as evident from Fig 8(B). In the initial docked pose, the Ligand_874 formed three H-bonds with residues Lys 439, Arg 478 and Gln 520. However, the ligand moved away during the MD simulations and only one H-bond with Arg478 was formed. The hydrophobic interactions of the ligand were also altered and reduced to three residues (Asp376, Gln379 and Gln520) out of the eight formed in the pre-MD initial docked pose (Fig 9).

### Substructure Fragment Analysis

To identify the structural fragments responsible for the activity of the active compounds, the inhibitors and non-inhibitors set was further searched for the presence of substructure fragments using Substructure fingerprint (SubFP). The substructures with a statistically significant p-value (p<0.01) were included in the study. The detailed analysis of the substructure fragments with their frequencies in the inhibitors and non-inhibitors is given in Table 4. Nitrate, carboxylic acid, Michael acceptor, alkene, anion and salt were highly significant among the inhibitors. It has been suggested that nitrates and their derivatives could be potential neuroprotective agents, with one such novel therapeutic agent, GT 1061, currently being in Phase I clinical trials [43]. The other patterns more frequent in inhibitors than non-inhibitors included vinylogous ester, vinylogous carbonyl, 1, 3-Tautomerizable, conjugated double bond, annelated rings and carbonic acid derivatives.

**Table 4:** Lists the names of predicted active drugs from DrugBank against Alzheimer’s by generated predictive models.

DrugBank ID	Drug name	Drug class	Glide Score (XP) (kcal/mol)	Glide energy (kcal/mol)	Literature reference
DB00171	Adenosine triphosphate	Dietary supplement	-9.34	-53.39	Coskuner and Murray, 2014
DB00158	Folic acid	Dietary supplement	-6.03	-50.97	Das, 2008, Ford et al., 2010
DB00144	Phosphatidylserine	Dietary supplement	-5.95	-45.36	Meng et al., 2014
DB00177	Valsartan	Antihypertensive	-3.92	-40.55	Wang et al., 2007
DB00328	Indomethacin	Non-steroidal anti-inflammatory drug	-3.50	-36.48	Jaturapatporn et al., 2012, Bernardi et al., 2012
DB00796	Candesartan	Antihypertensive	-3.08	-40.17	Anderson et al., 2011, Lithell et al., 2003
DB00307	Bexarotene	Oncology	-3.0	-23.90	Appleby et al., 2013
DB00966	Telmisartan	Antihypertensive	0.90	-41.03	Mogi et al., 2008

### Screening of the DrugBank Database

The predictive models generated in the present study were used to screen a set of 1,510 approved drugs downloaded from the DrugBank [44] database. The NB model predicted 600 drugs to be active, the RF model predicted 370 drugs to be active and the SMO model predicted 456 drugs to be active against the STEP enzyme in Alzheimer's. We conducted a consensus analysis, which resulted in 109 drugs predicted to be active by all the three models. The list of 109 drugs is available in S4 table.

### Novel Treatments for Alzheimer’s using Drug Repositioning

Drug repositioning, which is the process of identifying new therapeutic effects of existing medications, presents an excellent opportunity for the identification of better and safer treatments that would reduce the preclinical research required along with the time and costs involved in developing novel drugs [45]. The concept of drug repurposing has already been successful in treating many diseases including cancer, cardiovascular disease, obesity, irritable bowel syndrome, psychosis, smoking cessation, and Parkinson's disease [46, 47]. Drug repositioning has now been utilized in the development of AD treatments and has been reported in various studies [48, 49]. Corbett et al. detailed 15 drug candidates for repositioning in the case of AD, which included antihypertensives, antibiotics, anti-diabetic drugs and retinoid therapy. A total of 109 drugs were here predicted as active against Alzheimer’s and so could be used as repositioning candidates. However, we will be discussing those eight drugs for which any kind of study with respect to Alzheimer’s has already been reported. The 109 drugs predicted by our classification models included antihypertensives such as Valsartan [50], Telmisartan [51] and Candesartan [52, 53], which have previously been positioned as repurposing candidates for AD. The other drugs reported as active against STEP included Folic acid, Indomethacin, Adenosine triphosphate, Bexarotene and phosphatidylserine. Folic acid, a form of the water soluble Vitamin B9, has been reported to reduce the level of homocysteine in the blood, which is otherwise increased in cases of AD and may contribute to disease pathology [54, 55]. Non-steroidal anti-inflammatory drugs (NSAIDs) such as Indomethacin have also been found to slow the progression of the disease in Alzheimer’s patients by reducing the inflammatory action induced by Aβ [56, 57]. One of the early biomarkers in cases of AD is altered amyloid-β protein (Aβ) levels. In a recent study, ATP has been found to interact with Aβ and thus plays a possible role in the progression of the disease [58]. Phosphatidylserine has also been indicated in the treatment of Alzheimer's disease [59]. Various studies have reported that Bexarotene, commonly used to treat T-cell lymphomas, also affects apolipoprotein E induction and so could be a good candidate for Alzheimer’s treatment [60]. The details of the aforementioned drugs along with their classes are given in Table 5 and information concerning all the 109 drugs is given in S4 table.

**Table 5:** Shows the frequencies of substructure fragments in the inhibitors and non-inhibitors.

Substructure fragment number	Substructure name	Frequency in inhibitors	Frequency in non-inhibitors
SubFP187	Nitrate	325.17	0.19
SubFP84	Carboxylic acid	4.06	0.99
SubFP303	Michael acceptor	2.80	0.99
SubFP5	Alkene	2.09	0.99
SubFP297	Anion	2.00	0.99
SubFP299	Salt	2.00	0.99

To have a better understanding of the binding mechanisms as well as an independent evaluation approach, the eight repositioning candidates discussed above were screened against human protein tyrosine phosphatase, PTPN5 (STEP, striatum enriched phosphatase) (PDB ID: 2BV5). The drug molecules were first docked using Schrodinger’s Glide HTVS, after which the top scoring poses of all the drugs were docked using Glide’s XP docking strategy. We identified two high scoring repositioning candidates, namely Adenosine triphosphate (ATP) with a Glide score of -9.34 kcal/mol and Folic acid with a Glide score of -6.03 kcal/mol.

Adenosine triphosphate (ATP) formed six hydrogen bonds, out of which 2 hydrogen bonds were formed between the NH2 and NE atoms of active site residue Arg 478 and the O7 of ATP, with distances of 2.90 Å and 2.80 Å respectively. Two hydrogen bonds were formed between the NE2 atom of active site residue Gln 520 and the O5 and O6 atoms of ATP, with distances of 2.99 Å and 3.13 Å respectively. One hydrogen bond was formed between the NZ atom of Lys 439 and the O4 atom of ATP (3.02 Å) while the last bond was formed between the NE1 of Trp 435 and the O5 atom of the ligand, ATP (2.95 Å).

The second high scoring repositioning candidate was Folic acid, which formed six hydrogen bonds and had a Glide energy of -50.97 kcal/mol. It formed a hydrogen bond (H-bond) with the active site residue Arg 478 (2.81 Å) and other restudies, which involved Arg 303 (2.88 Å), Tyr 304 (2.68 Å), Trp 435 (2.95 Å), Lys 439 (3.04 Å) and Gln 520 (3.15 Å). The interacting residues for both the repositioning candidates, ATP and Folic acid, can be seen in Fig 3(C) and Fig 3(D) respectively. The chemical structures are shown in Fig 4(C) (ATP) and Fig 4(D) (Folic acid). Table 3 details the Glide score, the Glide energy, the interacting residues and the H-bond distances of the top scoring repositioning candidates.

### External Test Set Validation

The predictive models generated in the present study were used to screen the MyriaScreen diversity library of 23,165 compounds purchased from Sigma-Aldrich. The compounds were tested on the three models (NB, RF and SMO) generated using the BestFirst descriptors. The NB model predicted 7,285 compounds to be active, RF predicted 4,938 compounds to be active and SMO predicted 6,014 compounds that can act as inhibitors against STEP. The consensus between all three BestFirst models resulted in 1,226 compounds showing activity against STEP. The 1,226 compounds were further filtered for the presence of undesirable compounds using the SMARTsfilter web application. Out of the 1,226, 147 compounds did not contain any of the undesirable SMARTs patterns and passed through all the filters. The majority of the compounds did not pass through PAINS (51.9%), while 42.7% compounds did not pass through ALARM NMR and 37% compounds did not pass through Oprea. S5 table lists the final compounds from the MyriaScreen library that can play a possible role as STEP inhibitors.

The 147 compounds that passed the SMARTsfilter were further docked in the active site of human protein tyrosine phosphatase, PTPN5 (STEP, striatum enriched phosphatase) (PDB ID: 2BV5), using the Glide module of Schrodinger. The compounds were first docked using the HTVS approach followed by the XP docking approach. The top scoring compound, (3S)‐1‐(furan‐2‐ylmethyl)‐5‐oxopyrrolidine‐3-carboxylate (Ligand_5), had Glide score of -5.95 kcal/mol and Glide energy of -32.80 kcal/mol. The compound formed hydrogen bonds with three residues, one with active site residue Arg478 (2.68 Å) and the other two with Lys439 (2.59 Å) and Gln520 (3.26 Å) (Fig 3(B), Table 3). The residues involved in the hydrophobic interactions with the compound included Tyr304, Trp435, Scy472 and Gln516. The compound had a molecular weight of 290.20 g/mol, 1 donor hydrogen bond, 5.5 acceptor hydrogen bonds and a log P value of 0.5, which clearly indicate that the compound passed the Lipinski filter and so can be an effective inhibitor against Alzheimer’s. The chemical structure of the compound, (3S) ‐1‐ (furan‐2‐ylmethyl) ‐5‐oxopyrrolidine‐3-carboxylate (Ligand_5), is shown in Fig 4(B).

## Conclusion

Alzheimer’s disease is a major health concern globally, particularly among the elderly. AD affects millions of lives worldwide in addition to the social and economic burdens associated with the disease. Owing to the limited number of therapeutic options currently available, along with low drug efficacy, it has become crucial to develop reasonably priced, safe, secure and effectual anti-Alzheimer’s drugs. Drug discovery and development is an extremely slow, costly process and is prone to a high rate of failures. The high-throughput screens have resulted in a large amount of freely available data, which offers tremendous prospects. However, prioritizing the molecules before experimental screening in the drug discovery process still remains an issue to be addressed.

The present study deals with the generation of machine learning based computational models, which can prioritize molecules based on their chemical properties and activities. The machine learning models were generated using 154 2D molecular descriptors identified via the Naive Bayes, Random Forest and Sequential Minimization Algorithm methods. In addition, we used the BestFirst search algorithm to identify relevant descriptors, which resulted in 10 descriptors. The models generated at both descriptor levels performed extremely well in classifying inhibitors and non-inhibitors against striatal-enriched protein tyrosine phosphatase (STEP), a tyrosine phosphatase reported to be associated with AD. Until now, the proposed strategy has been applied to develop inhibitors against Acetyl cholinesterase (Ache) [61]. However, since no effective drug targeting Ache has been reported so far, the identification of novel inhibitors against other crucial targets can be considered as a viable and worthy approach.

The compounds predicted to be active by the machine learning based models were screened using molecular docking studies for deeper insights into their binding mechanisms. We identified two top scoring compounds: Ligand_874 (AID 588621) with a Glide score of -8.11 kcal/mol and Ligand_7 (Sigma_Aldrich compounds) with a Glide score of -5.95 kcal/mol. We also performed MD simulation studies for the docked complex of Ligand_874 and STEP. This stabilized the docked complex and resulted in stronger and energetically more favorable interactions as compared to the pre-MD docked complex. Our models also predicted some of the FDA approved drugs that can be used as repositioning candidates in cases of Alzheimer’s. The two top scoring drug candidates that can be repositioned and used against Alzheimer’s were ATP and Folic acid, with Glide scores of -9.34 kcal/mol and -6.03 kcal/mol respectively.

To identify the structural fragments contributing to the activity of the molecules, we used a substructure pattern recognition method in which the substructure fingerprints were generated based on a predefined fingerprint dictionary. We computed the frequencies of the substructures in the inhibitor and non-inhibitor sets and found that substructures like nitrate, carboxylic acid, Michael acceptor, alkene, anion and salt were present in a large number in the active compound sets. The proposed computational pre-screening approach would improve the likelihood of identifying actives from a large number of unscreened compounds, which could potentially result in less resource intensive and more cost effective drugs that will provide better healthcare options to patients.

## Supporting Information

S1 Table Details of the parameters used in generating the models using NB, RF and SMO machine learning algorithms. (DOCX)

S2 Table List of the 154 non-redundant attributes and 10 BestFirst descriptors. (DOCX)

S3 Table List of performance of the classifiers using 5-fold cross validation. (DOCX)

S4 Table List of 109 drugs predicted as active by all the three models NB, RF and SMO. (DOCX)

S5 Table List of 147 final compounds from MyriaScreen library which can have a possible role as STEP inhibitors. (DOCX)
