# Supplementary material for: Checking the STEP-Associated Trafficking and Internalization of Glutamate Receptors for Reduced Cognitive Deficits: A Machine Learning Approach-Based Cheminformatics Study and Its Application for Drug Repurposing
Source: PLoS One. 2015 Jun 12;10(6):e0129370. doi: 10.1371/journal.pone.0129370 (PMC4466797; doi:10.1371/journal.pone.0129370)
Supplement: S1 Table — (DOCX) [file pone.0129370.s001.docx]

| ***Model*** | ***Parameters*** |
| --- | --- |
| Naïve Bayes | Debug: False  Display model in old format: False  Use kernel estimator: False  Use supervised discretization: False |
| Random Forest | Debug: False  Maximum depth of trees (maxDepth) : 0 (unlimited)  Number of attributes (numFeatures) : 0  Number of trees to be generated (numTrees): 10  Seed: 1 |
| SMO | Build logistic models: False  Complexity parameter, c: 1.0  Checks turned off: False  Debug: False  Epsilon: 1.0 E -12  Filter type: Normalized training data  Kernel : Polykernel  Number of folds: -1  Random seeds: 1  Tolerance parameter: 0.001 |
