# Supplementary material for: Checking the STEP-Associated Trafficking and Internalization of Glutamate Receptors for Reduced Cognitive Deficits: A Machine Learning Approach-Based Cheminformatics Study and Its Application for Drug Repurposing
Source: PLoS One. 2015 Jun 12;10(6):e0129370. doi: 10.1371/journal.pone.0129370 (PMC4466797; doi:10.1371/journal.pone.0129370)
Supplement: S2 Table — (DOCX) [file pone.0129370.s002.docx]

| **RemoveUseless attributes (154)** | | | **BestFirst descriptors (10)** | | |
| --- | --- | --- | --- | --- | --- |
| **Pharmacophore fingerprints (122)** | **Weighted burden number (24)** | **Property (8)** | **Pharmacophore fingerprints (6)** | **Weighted burden number (4)** | **Property (0)** |
| NEG_01_NEG- NEG_07_NEG | WBN_GC_L_0.25 | XLogP | NEG_06_NEG | WBN_GC_H_0.25 |  |
| NEG_03_POS- NEG_07_POS | WBN_GC_H_0.25 | PSA | NEG_04_HBD | WBN_GC_L_0.50 |  |
| NEG_01_HBD- NEG_07_HBD | WBN_GC_L_0.50 | NumRot | NEG_05_HBD | WBN_GC_L_1.00 |  |
| NEG_03_HBA- NEG_07_HBA | WBN_GC_H_0.50 | NumHBA | NEG_02_ARC | WBN_EN_H_0.25 |  |
| NEG_02_ARC- NEG_07_ARC | WBN_GC_L_0.75 | NumHBD | POS_03_HBD |  |  |
| NEG_02_HYP- NEG_07_HYP | WBN_GC_H_0.75 | MW | HBD_06_HBD |  |  |
| POS_03_POS- POS_07_POS | WBN_GC_L_1.00 | BBB |  |  |  |
| POS_02_HBD- POS_07_HBD | WBN_GC_H_1.00 | BadGroup |  |  |  |
| POS_03_HBA- POS_07_HBA | WBN_EN_L_0.25 |  |  |  |  |
| POS_02_ARC- POS_07_ARC | WBN_EN_H_0.25 |  |  |  |  |
| POS_02_HYP- POS_07_HYP | WBN_EN_L_0.50 |  |  |  |  |
| HBD_03_HBD- HBD_07_HBD | WBN_EN_H_0.50 |  |  |  |  |
| HBD_03_HBA- HBD_07_HBA | WBN_EN_L_0.75 |  |  |  |  |
| HBD_02_ARC- HBD_07_ARC | WBN_EN_H_0.75 |  |  |  |  |
| HBD_02_HYP- HBD_07_HYP | WBN_EN_L_1.00 |  |  |  |  |
| HBA_03_HBA- HBA_07_HBA | WBN_EN_H_1.00 |  |  |  |  |
| HBA_03_ARC- HBA_07_ARC | WBN_LP_L_0.25 |  |  |  |  |
| HBA_02_HYP- HBA_07_HYP | WBN_LP_H_0.25 |  |  |  |  |
| ARC_01_ARC- ARC_07_ARC | WBN_LP_L_0.50 |  |  |  |  |
| ARC_02_HYP- ARC_07_HYP | WBN_LP_H_0.50 |  |  |  |  |
| HYP_01_HYP- HYP_07_HYP | WBN_LP_L_0.75 |  |  |  |  |
|  | WBN_LP_H_0.75 |  |  |  |  |
|  | WBN_LP_L_1.00 |  |  |  |  |
|  | WBN_LP_H_1.00 |  |  |  |  |
