# Supplementary material for: Checking the STEP-Associated Trafficking and Internalization of Glutamate Receptors for Reduced Cognitive Deficits: A Machine Learning Approach-Based Cheminformatics Study and Its Application for Drug Repurposing
Source: PLoS One. 2015 Jun 12;10(6):e0129370. doi: 10.1371/journal.pone.0129370 (PMC4466797; doi:10.1371/journal.pone.0129370)
Supplement: S3 Table — (DOCX) [file pone.0129370.s003.docx]

| **For 154 attributes (RemoveUseless filter)** | | | | | | | | | |
| --- | --- | --- | --- | --- | --- | --- | --- | --- | --- |
| **Model** | **Cross-validation** | **True Positives** | **True Negatives** | **False Positives** | **False Negatives** | **Sensitivity (%)** | **Specificity (%)** | **Accuracy** | **G-mean** |
| ***Naive bayes*** | 2 | 92 | 57215 | 14454 | 85 | 52 | 79.8 | 79.7 | 0.64 |
|  | 3 | 92 | 57215 | 14454 | 85 | 52 | 79.8 | 79.7 | 0.64 |
|  | 4 | 92 | 57215 | 14454 | 85 | 52 | 79.8 | 79.7 | 0.64 |
|  | 5 | 92 | 57215 | 14454 | 85 | 52 | 79.8 | 79.7 | 0.64 |
| ***Random forest*** | 2 | 115 | 56697 | 14972 | 62 | 64.9 | 79.1 | 79 | 0.71 |
|  | 3 | 115 | 56697 | 14972 | 62 | 64.9 | 79.1 | 79 | 0.71 |
|  | 4 | 115 | 56697 | 14972 | 62 | 64.9 | 79.1 | 79 | 0.71 |
|  | 5 | 115 | 56697 | 14972 | 62 | 64.9 | 79.1 | 79 | 0.71 |
| ***SMO*** | 2 | 123 | 56903 | 14766 | 54 | 69.4 | 79.3 | 79.3 | 0.74 |
|  | 3 | 123 | 56903 | 14766 | 54 | 69.4 | 79.3 | 79.3 | 0.74 |
|  | 4 | 123 | 56903 | 14766 | 54 | 69.4 | 79.3 | 79.3 | 0.74 |
|  | 5 | 123 | 56903 | 14766 | 54 | 69.4 | 79.3 | 79.3 | 0.74 |
|  |  |  |  |  |  |  |  |  |  |
| **For selected 34 attributes** | | | | | | | | | |
| **Model** | **Cross-validation** | **True Positives** | **True Negatives** | **False Positives** | **False Negatives** | **Sensitivity (%)** | **Specificity (%)** | **Accuracy** | **MCC** |
| ***Naive bayes*** | 2 | 58 | 57588 | 14081 | 119 | 32.7 | 80.3 | 80.2 | 0.51 |
|  | 3 | 58 | 57588 | 14081 | 119 | 32.7 | 80.3 | 80.2 | 0.51 |
|  | 4 | 58 | 57588 | 14081 | 119 | 32.7 | 80.3 | 80.2 | 0.51 |
|  | 5 | 58 | 57588 | 14081 | 119 | 32.7 | 80.3 | 80.2 | 0.51 |
| ***Random forest*** | 2 | 102 | 58049 | 13620 | 75 | 57.6 | 80.9 | 80.9 | 0.68 |
|  | 3 | 102 | 58049 | 13620 | 75 | 57.6 | 80.9 | 80.9 | 0.68 |
|  | 4 | 102 | 58049 | 13620 | 75 | 57.6 | 80.9 | 80.9 | 0.68 |
|  | 5 | 102 | 58049 | 13620 | 75 | 57.6 | 80.9 | 80.9 | 0.68 |
| ***SMO*** | 2 | 65 | 57796 | 13873 | 112 | 36.7 | 80.6 | 80.6 | 0.54 |
|  | 3 | 65 | 57796 | 13873 | 112 | 36.7 | 80.6 | 80.6 | 0.54 |
|  | 4 | 65 | 57796 | 13873 | 112 | 36.7 | 80.6 | 80.6 | 0.54 |
|  | 5 | 65 | 57796 | 13873 | 112 | 36.7 | 80.6 | 80.6 | 0.54 |
