# Supplementary material for: Checking the STEP-Associated Trafficking and Internalization of Glutamate Receptors for Reduced Cognitive Deficits: A Machine Learning Approach-Based Cheminformatics Study and Its Application for Drug Repurposing
Source: PLoS One. 2015 Jun 12;10(6):e0129370. doi: 10.1371/journal.pone.0129370 (PMC4466797; doi:10.1371/journal.pone.0129370)
Supplement: S4 Table — (DOCX) [file pone.0129370.s004.docx]

| **List of 109 drugs predicted active by the predictive models** | |
| --- | --- |
| **Drug name** | **DrugBank ID** |
| Acamprosate | DB00659 |
| Adapalene | DB00210 |
| Adefovir Dipivoxil | DB00718 |
| Adenosine monophosphate | DB00131 |
| Adenosine triphosphate | DB00171 |
| Alitretinoin | DB00523 |
| Amiloride | DB00594 |
| Amlexanox | DB01025 |
| Benzthiazide | DB00562 |
| Bexarotene | DB00307 |
| Bicalutamide | DB01128 |
| Brimonidine | DB00484 |
| Bumetanide | DB00887 |
| Calcium Gluceptate | DB00326 |
| Candesartan | DB00796 |
| Cefaclor | DB00833 |
| Cefadroxil | DB01140 |
| Cefdinir | DB00535 |
| Cefixime | DB00671 |
| Cefmetazole | DB00274 |
| Cefprozil | DB01150 |
| Ceftazidime | DB00438 |
| Ceftriaxone | DB01212 |
| Cefuroxime | DB01112 |
| Cephalexin | DB00567 |
| Ceruletide | DB00403 |
| Chlordiazepoxide | DB00475 |
| Cimetidine | DB00501 |
| Colistimethate | DB01111 |
| Cromoglicic acid | DB01003 |
| Dactinomycin | DB00970 |
| Demecarium | DB00944 |
| Dextrothyroxine | DB00509 |
| Diatrizoate | DB00271 |
| Diflunisal | DB00861 |
| Doxacurium chloride | DB01135 |
| Edrophonium | DB01010 |
| Enoxacin | DB00467 |
| Eprosartan | DB00876 |
| Eszopiclone | DB00402 |
| Famotidine | DB00927 |
| Felbamate | DB00949 |
| Flavoxate | DB01148 |
| Folic Acid | DB00158 |
| Fosamprenavir | DB01319 |
| Foscarnet | DB00529 |
| Fosphenytoin | DB01320 |
| Furosemide | DB00695 |
| Gadobenate Dimeglumine | DB00743 |
| Gatifloxacin | DB01044 |
| Gemifloxacin | DB01155 |
| Indomethacin | DB00328 |
| Isotretinoin | DB00982 |
| Lactulose | DB00581 |
| L-Carnitine | DB00583 |
| L-Cysteine | DB00151 |
| Leucovorin | DB00650 |
| Levofloxacin | DB01137 |
| Levothyroxine | DB00451 |
| Liothyronine | DB00279 |
| Lomefloxacin | DB00978 |
| L-Phenylalanine | DB00120 |
| L-Serine | DB00133 |
| L-Threonine | DB00156 |
| L-Tryptophan | DB00150 |
| Lymecycline | DB00256 |
| Meclofenamic acid | DB00939 |
| Mefenamic acid | DB00784 |
| Melphalan | DB01042 |
| Meropenem | DB00760 |
| Mesalazine | DB00244 |
| Methantheline | DB00940 |
| Methotrexate | DB00563 |
| Mezlocillin | DB00948 |
| Micafungin | DB01141 |
| Mimosine | DB01055 |
| Montelukast | DB00471 |
| Moricizine | DB00680 |
| Moxifloxacin | DB00218 |
| N-Acetyl-D-glucosamine | DB00141 |
| Nedocromil | DB00716 |
| Niacin | DB00627 |
| Novobiocin | DB01051 |
| Ofloxacin | DB01165 |
| Olmesartan | DB00275 |
| Oxaprozin | DB00991 |
| Pemetrexed | DB00642 |
| Penicillamine | DB00859 |
| Pentosan Polysulfate | DB00686 |
| Phenprocoumon | DB00946 |
| Phosphatidylserine | DB00144 |
| Porfimer | DB00707 |
| Probenecid | DB01032 |
| Propantheline | DB00782 |
| Riboflavin | DB00140 |
| Sparfloxacin | DB01208 |
| Streptozocin | DB00428 |
| Succimer | DB00566 |
| Sulfoxone | DB01145 |
| Telmisartan | DB00966 |
| Tetrahydrofolic acid | DB00116 |
| Tolmetin | DB00500 |
| Treprostinil | DB00374 |
| Tretinoin | DB00755 |
| Valsartan | DB00177 |
| Vancomycin | DB00512 |
| Warfarin | DB00682 |
| Zoledronate | DB00399 |
| Zopiclone | DB01198 |
