# Supplementary material for: Checking the STEP-Associated Trafficking and Internalization of Glutamate Receptors for Reduced Cognitive Deficits: A Machine Learning Approach-Based Cheminformatics Study and Its Application for Drug Repurposing
Source: PLoS One. 2015 Jun 12;10(6):e0129370. doi: 10.1371/journal.pone.0129370 (PMC4466797; doi:10.1371/journal.pone.0129370)
Supplement: S5 Table — (DOCX) [file pone.0129370.s005.docx]

**List of 147 final compounds from MyriaScreen library which can have a possible role as STEP inhibitors.**

C(=O)(c1c2ccc(c1)CCc1ccc(c(C(=O)[O-])c1)CC2)[O-]

c1(nc2c(o1)cccc2)[C@H]1C[NH2+]CCC1

c1(nc2c(o1)cccc2)[C@@H]1C[NH2+]CCC1

n12c(nnc1)ccc(n2)NCCC[NH+]1CCOCC1

c1cc2ccc1CCc1ccc(c(c1)C(=O)[O-])CC2

O=c1n(c(=O)c2n[nH]nc2n1C)C

O=c1n(c(=O)c2nn[nH]c2n1C)C

O=c1n(c(=O)c2[nH]nnc2n1C)C

c1(nn(c2c1CCCC2)C)C(=O)[O-]

c1(c(cc2c(c1)nc[nH]2)C(=O)[O-])C(=O)[O-]

c1(c(cc2c(c1)[nH]cn2)C(=O)[O-])C(=O)[O-]

C1CCC[NH+]1CC[C@](c1ccc(cc1)C)(c1ccccc1)O

C1CCC[NH+]1CC[C@@](c1ccc(cc1)C)(c1ccccc1)O

c1(CNC(=O)CCCC(=O)[O-])ccco1

[C@H]1([C@@H]2C[C@@H]([C@H]1C(=O)[O-])C=C2)C(=O)NCc1ccoc1

[C@@H]1([C@@H]2C[C@@H]([C@H]1C(=O)[O-])C=C2)C(=O)NCc1ccoc1

[C@H]1([C@H]2C[C@H]([C@H]1C(=O)[O-])C=C2)C(=O)NCc1ccoc1

[C@@H]1([C@H]2C[C@H]([C@H]1C(=O)[O-])C=C2)C(=O)NCc1ccoc1

[C@H]1([C@@H]2C[C@@H]([C@@H]1C(=O)[O-])C=C2)C(=O)NCc1ccoc1

[C@@H]1([C@@H]2C[C@@H]([C@@H]1C(=O)[O-])C=C2)C(=O)NCc1ccoc1

[C@H]1([C@H]2C[C@H]([C@@H]1C(=O)[O-])C=C2)C(=O)NCc1ccoc1

[C@@H]1([C@H]2C[C@H]([C@@H]1C(=O)[O-])C=C2)C(=O)NCc1ccoc1

c1(cc2c(cc1C(=O)[O-])nc[nH]2)C

c1(cc2c(cc1C(=O)[O-])[nH]cn2)C

C(c1ccccc1)(c1ccccc1)(CC[N@@H+]1CC[C@](CC1)(c1ccc(cc1)Cl)O)C(=O)N(C)C

c12c(cc(n(c1=O)C)CCCC(=O)[O-])cccc2

c1(oc(cc1)C)C(=O)NCCC(=O)[O-]

c1(c(c(oc1C)C)C(=O)[O-])C(=O)[O-]

C(=O)(c1cc(=O)n(c(=O)[nH]1)C)[O-]

C1CCCC(C1)n1c(=O)[nH][nH]c1=O

c1ccc2c(c1)c(=O)[nH]c(=O)n2C

[C@H]12C(=CC[C@@H](C1(C)C)C2)CC[NH+](C)C

[C@@H]12C(=CC[C@H](C1(C)C)C2)CC[NH+](C)C

c12c(CC[C@@H](C1)CCC(=O)[O-])cccc2

c12c(CC[C@H](C1)CCC(=O)[O-])cccc2

[nH]1c2c(c(c1C(=O)[O-])CCCC(=O)[O-])cccc2

C1(CC(c2ccccc2)(c2ccccc2)O)(CCCCC1)[NH+](C)C

c12c(c(c(=O)[nH]c1cccc2)C)C(=O)[O-]

O=c1[nH]c2c(nc1)n(c(=O)n(c2=O)C)C

c1(nc(oc1NCCC[NH+]1CCOCC1)[C@H]1CC[C@@H](CC1)CCCC)C#N

c12c([C@@H](C(=C(N)N1)C#N)c1ccc(cc1)Cl)nc(C(=O)OCC)o2

c12c([C@H](C(=C(N)N1)C#N)c1ccc(cc1)Cl)nc(C(=O)OCC)o2

c12CCCn3c1c(c(c(c3=O)C(=O)NCc1ccco1)O)ccc2

c12CCCn3c1c(c(c(c3=O)C(=O)Nc1ccccc1)O)ccc2

c12CCCn3c1c(c(c(c3=O)C(=O)Nc1cccnc1)O)ccc2

N1(C(=O)C[C@H](C1)C(=O)[O-])Cc1occc1

N1(C(=O)C[C@@H](C1)C(=O)[O-])Cc1occc1

c12c(ncn(c1=O)C1CCCCC1)oc(c2C(=O)[O-])C

c1(c(oc(n1)C)OCC)C(=O)[O-]

c12CCCn3c1c(c(c(c3=O)C(=O)Nc1ncccc1)O)ccc2

c12CCCn3c1c(c(c(c3=O)C(=O)Nn1c(nc3c(c1=O)cccc3)C)O)ccc2

c1(c(C)cc(C)cc1C)NC(=O)CCC[NH+]1CCN(CC1)C

c1(c(C)cc(C)cc1C)NC(=O)CCCN1CC[NH+](CC1)C

c1(c(C)cc(C)cc1C)NC(=O)CCC[N@@H+]1CC[N@H+](CC1)C

C(=O)(c1c(C(=O)[O-])cccc1)N1CCCCC1

c12c(n(cc(c1=O)C(=O)[O-])CC)nc(nc2C)C

c12c(n(c(=O)n(c1=O)C)C)nc(n2CCCCC)CN1CCOCC1

c12c(n(c(=O)n(c1=O)C)C)[NH2+]C(=N2)C[NH+](C)C

c12c(n(c(=O)n(c1=O)C)C)nc([nH]2)CN1CCN(CC1)C

c12c(n(c(=O)n(c1=O)C)C)[nH]c(n2)CN1CCN(CC1)C

c12c(n(c(=O)n(c1=O)C)C)[NH2+]C(=N2)CN1CCN(CC1)C

c12c(n(c(=O)n(c1=O)C)C)[NH2+]C(=N2)C[NH+]1CCN(CC1)C

c12c(n(c(=O)n(c1=O)C)C)nc([nH]2)CN1CC[NH+](CC1)C

c12c(n(c(=O)n(c1=O)C)C)[nH]c(n2)CN1CC[NH+](CC1)C

c12c(n(c(=O)n(c1=O)C)C)[NH2+]C(=N2)CN1CC[N@@H+](CC1)C

c12c(n(c(=O)n(c1=O)C)C)[NH2+]C(=N2)C[N@@H+]1CC[N@@H+](CC1)C

c12c(n(c(=O)n(c1=O)C)C)nc(n2CC)CN1CCOCC1

C1OCC[NH+](C1)CCCNC(=O)c1cc(c(cc1)Cl)Cl

c12c(n(cc(c1=O)C(=O)[O-])C1CC1)cc(c(c2)F)F

C(=O)(C(c1ccccc1)c1ccccc1)NCCC[NH+]1CCOCC1

n1(c(=O)n(c2c(c1=O)n(c(n2)N(C)C)CC(=C)C)C)C

n1(c(=O)n(c2c(c1=O)n1c(n2)n(c(c1)c1ccccc1)c1ccccc1)C)C

C(=O)(c1c(cccc1)NC(=O)C(C)(C)C)N1CC[NH+](CC1)C

O1COc2c1ccc(c2)/C=C(\C#N)/c1ccc(cc1)Cl

O1COc2c1ccc(c2)/C=C(\C#N)/c1cc(ccc1)C

O1COc2c1ccc(c2)/C=C(\C#N)/c1ccccc1Cl

c12c(n(c(=O)n(c1=O)C)C)nc(n2C)OCC

n1(c(=O)n(c2c(c1=O)n(c(n2)OC(C)C)C)C)C

n1(c(=O)n(c2c(c1=O)n(c(n2)N1CCCCC1)Cc1c2c(ccc1)cccc2)C)C

[nH]1c(=O)n(c2c(c1=O)n1c(n2)n(c(c1)C)CCc1ccccc1)C

n1nc([nH][nH]c1C(=O)[O-])C(=O)[O-]

o1c(ccc1C(c1oc(cc1)C(=O)[O-])(C)C)C(=O)[O-]

c12c(nc3n1c(cc(c3C#N)CCC)N1CCN(CC1)C(=O)c1occc1)cccc2

n1(ncc2c1ncnc2N1CC[NH+](CC1)C)Cc1ccc(cc1)Cl

n1(ncc2c1ncnc2N1CC[NH+](CC1)C)Cc1ccc(cc1)C

c12c(OC(=C([C@@H]1c1cc3c(cc1)OCO3)C#N)N)cc([nH]c2=O)C

c12c(OC(=C([C@@H]1c1cc3c(cc1)OCO3)C#N)N)cc([nH]c2=O)C

c12CCCn3c1c(c(c(c3=O)C(=O)Nc1ccncc1)O)ccc2

c12CCCn3c1c(c(c(c3=O)C(=O)Nc1ncccn1)O)ccc2

n1(c(=O)n(c2c(c1=O)n(c(n2)OCCC)CCC)C)C

[nH]1c(=O)n(c2c(c1=O)n(c(n2)NC1CCCCC1)CCCC)C

[nH]1c(=O)n(c2c(c1=O)n(c(n2)N1CCCC1)C)C

[nH]1c(=O)n(c2c(c1=O)n(c(n2)NCc1occc1)C)C

c12c(nc(n1CCCc1ccccc1)NC)n(c(=O)n(c2=O)C)C

c12c(nc([nH]1)NCCCO)n(c(=O)[nH]c2=O)C

[C@@H]1([C@H]([C@@H]([C@H]1c1ccccc1)C(=O)NCCC[NH+]1CCCC1)c1ccccc1)C(=O)NCCC[NH+]1CCCC1

c12c(nc(n1CCCCC)N1CC[NH+](CC1)CC)n(c(=O)[nH]c2=O)C

c12c(nc(n1CC(=C)C)NCCCn1cncc1)n(c(=O)[nH]c2=O)C

c12c(nc(n1CC(=C)C)NCCC[NH3+])n(c(=O)[nH]c2=O)C

c12c(nc([nH]1)N1CC[NH+](CC1)c1ccccc1)n(c(=O)[nH]c2=O)C

c12c([nH]c(n1)N1CC[NH+](CC1)c1ccccc1)n(c(=O)[nH]c2=O)C

c12c(n(c(=O)[nH]c1=O)C)[NH2+]C(=N2)N1CC[NH+](CC1)c1ccccc1

n1(c(=O)n(c2c(c1=O)n(c(n2)NCC)Cc1ccc(cc1)C)C)C

[nH]1c(=O)n(c2c(c1=O)n(c(n2)N(Cc1ccccc1)C)CCC)C

c1(c(c(cc(c1C)C)C)C)C(=O)[O-]

c1(c(c(c(C)[nH]1)C(=O)OCc1ccccc1)C)C(=O)[O-]

[nH]1c(=O)n(c2c(c1=O)n(c(n2)NC)CC(=C)C)C

[nH]1c(=O)n(c2c(c1=O)n(c(n2)NCC)CC(=C)C)C

n1(c(=O)n(c2c(c1=O)n(c(n2)N(C)C)Cc1ccc(cc1)C)C)C

o1c2c(c3c1cc[nH]c3=O)cccc2

n1(c(=O)n(c2c(c1=O)n(c(n2)C#CCO)C)C)C

c1(ccc(cc1)F)c1cc(ncn1)C(=O)[O-]

[nH]1c(=O)n(c2c(c1=O)n(cn2)CCC)C

n1(c(=O)n(c2c(c1=O)N=C([NH+]2C)[N+](=O)[O-])C)C

c1c2c(cc(c1)c1nnn[nH]1)OCO2

c1c2c(cc(c1)c1nn[nH]n1)OCO2

c1c2c(cc(c1)c1nn[n-]n1)OCO2

n1(ncc(c1C)C(=O)[O-])c1ccc(cc1)F

c1cc(ccc1n1c(cc(n1)c1ccc(cc1)F)C(=O)[O-])Cl

[nH]1nc(c(c1)C(=O)[O-])c1ccc(cc1)F

n1[nH]c(c(c1)C(=O)[O-])c1ccc(cc1)F

[nH]1nc(cc1c1ccc(cc1)Cl)C(=O)[O-]

n1[nH]c(cc1c1ccc(cc1)Cl)C(=O)[O-]

o1nc(cc1C(=O)[O-])c1ccc(cc1)F

c12c(c(=O)n(c(=O)n1C)C)N=C(C[N@H+]1[C@H](C)CCCC1)[NH2+]2

c12c(c(=O)n(c(=O)n1C)C)N=C(C[N@@H+]1[C@@H](C)CCCC1)[NH2+]2

c12c(c(=O)n(c(=O)n1C)C)N=C(C[N@@H+]1[C@H](C)CCCC1)[NH2+]2

c12c(c(=O)n(c(=O)n1C)C)N=C(C[N@@H+]1[C@@H](C)CCCC1)[NH2+]2

n1(c(=O)n(c2c(c1=O)n(c(n2)OCCC)CC)C)C

c12c(c(=O)n(c(=O)n1C)C)n(c(n2)CN1CCN(CC1)C)CCCCC

c12c(c(=O)n(c(=O)n1C)C)n(c(n2)CN1CC[NH+](CC1)C)CCCCC

c12c(c(=O)n(c(=O)n1C)C)N=C(C[N@@H+]1C[C@@H](CCC1)C)[NH2+]2

c12c(c(=O)n(c(=O)n1C)C)N=C(C[N@@H+]1C[C@H](CCC1)C)[NH2+]2

c12c(c(=O)n(c(=O)n1C)C)N=C(C[N@@H+]1C[C@@H](CCC1)C)[NH2+]2

[nH]1c(=O)n(c2c(c1=O)n(c(n2)NCc1occc1)CCCC)C

c12c(c(=O)n(c(=O)n1C)C)N=C(C[N@@H+]1C[C@H](CCC1)C)[NH2+]2

c12c(n(c(=O)n(c1=O)C)C)nc(n2CC(C)C)CN1CCOCC1

n1(c(=O)n(c2c(c1=O)n(c(n2)N1CCOCC1)CCC#N)C)C

n1(c(=O)n(c2c(c1=O)n(c(n2)NC1CCCCC1)CCC#N)C)C

[nH]1c(=O)n(c2c(c1=O)n(c(n2)N1CCCCCC1)C)C

c1(c2ccccc2)[nH]c(cc1)C(=O)[O-]

c12c(nc(n1CC(=C)C)NCCC)n(c(=O)n(c2=O)C)C

c12c(nc(n1CC(=C)C)N1CC[NH+](CC1)CC)n(c(=O)[nH]c2=O)C

c12c(nc([nH]1)NCCCn1cncc1)n(c(=O)[nH]c2=O)C

c12c(nc([nH]1)NCCCN1CCOCC1)n(c(=O)[nH]c2=O)C

c12c(nc([nH]1)NCCC[NH+]1CCOCC1)n(c(=O)[nH]c2=O)C

n1(c(=O)c2c(c3c(c1=O)cccc3)cccc2)c1ccc(C(=O)[O-])cc1
